# Exercise Impacts Liver Disease: Balancing Metabolic and Immune Homeostasis

**DOI:** 10.7150/ijbs.126841

**Published:** 2026-04-03

**Authors:** Chen Cheng, Wentao Xu, Shanshan Wu, Jia Gao, Jianshang Huang, Yunsheng Dong, Jingjing Ji, Li Zuo, Hua Wang

**Affiliations:** 1 School of Pharmacy, Anhui Medical University, Hefei, 230022, China.; 2 Department of Oncology, the First Affiliated Hospital of Anhui Medical University, Hefei, 230022, China.; 3 Innovation and Entrepreneurship Laboratory for College Students, Anhui Medical University, Hefei, 230032, China.; 4 Department of Gastroenterology, The First Affiliated Hospital of Anhui Medical University, Hefei, 230022, China.; 5 Laboratory of Molecular Biology, and Department of Biochemistry, School of Basic Medical Science, Anhui Medical University, Hefei, 230032, China.; 6 Center for Experimental Animals, Anhui Medical University, Hefei, 230032, China.

**Keywords:** liver diseases, exercise intervention, molecular pathways, metabolic regulation, immune regulation

## Abstract

Liver diseases pose a significant global health burden. This review systematically elucidates the crucial role of exercise as a non-pharmacological intervention in the prevention and treatment of various liver conditions, including metabolic dysfunction-associated steatotic liver disease (MASLD), alcohol-related liver disease (ALD), viral hepatitis, liver fibrosis, cirrhosis, and hepatocellular carcinoma (HCC). Exercise effectively delays disease progression and improves patients' quality of life through multi-targeted mechanisms, such as improving glucose and lipid metabolism, enhancing insulin sensitivity, regulating immune-inflammatory responses, inhibiting hepatic stellate cell activation, and remodeling the tumor microenvironment. Future research should focus on developing individualized, precise exercise prescriptions and further exploring its molecular mechanisms by integrating multi-omics technologies, thereby providing innovative strategies for the comprehensive management of liver diseases.

## 1. Introduction

As a core organ of the human body, the liver plays a key role in energy metabolism, protein synthesis, and toxin excretion. Liver diseases are primarily characterized by pathological features such as hepatocyte injury, inflammatory cell infiltration, and activation of hepatic stellate cells. The persistent progression of these conditions can lead to impaired liver function and structural damage 1. Globally, nearly 2 million people die from liver diseases each year, accounting for approximately 4% of total global mortality 2. Among these, metabolic dysfunction-associated steatotic liver disease (MASLD) and alcohol-related liver disease (ALD) have a large patient base, with incidence rates continuing to rise 3, 4. Without timely intervention, these conditions may progress to end-stage liver diseases such as cirrhosis and hepatocellular carcinoma (HCC) 2. Therefore, early intervention is crucial for controlling the progression of liver diseases and alleviating the burden on healthcare systems. Studies have shown that regular exercise not only effectively prevents and treats various liver diseases 4-9, but also reduces the risk of liver cancer and improves the quality of life in patients with chronic liver diseases through multiple mechanisms, including improving lipid profiles, regulatory myokines, maintaining muscle mass, and increasing insulin sensitivity 10. In summary, the necessity of studying exercise lies in its multi-target effects, such as regulating metabolism, inhibiting hepatocyte inflammation, and reducing fibrosis, thereby safely, economically, and non-pharmacologically delaying the progression of liver diseases and effectively improving patients' quality of life.

This review summarizes the role and related mechanisms of exercise in the prevention and treatment of liver diseases, discussing the positive effects of exercise on MASLD, cirrhosis, and HCC [Table 1]. It aims to elucidate how exercise, through multiple mechanisms such as improving glucose and lipid metabolism, enhancing insulin sensitivity, and modulating the immune system, not only reduces the risk of liver diseases and improves the quality of life in patients with chronic liver diseases and liver transplant recipients, but also provides a scientific basis for the comprehensive management of liver diseases.

## 2. Definition and classification of exercise

Physical activity refers to bodily movement that effectively increases metabolic rate and energy expenditure, with its intensity quantifiable and graded using metabolic equivalents (METs): light (1.6 < 3.0 METs), moderate (3 < 6 METs), and vigorous (6.0-9.0 METs). Assessment can also be based on heart rate reserve or percentage of maximal heart rate (%HRmax), roughly corresponding to < 55%, 55 < 70%, and 70-90% HRmax, respectively. An exercise prescription should comprehensively consider exercise mode, frequency, duration, total energy expenditure, as well as activity structure and characteristics of the energy supply systems 11.

Aerobic exercise primarily relies on aerobic metabolism for energy, characterized by moderate intensity and sustainability, such as running, brisk walking, cycling, and swimming. It improves liver function through multiple pathways: enhancing insulin sensitivity 12, 13, inhibiting hepatic gluconeogenesis 13, reducing the deposition of free fatty acids (FFAs) in the liver 12, 13, and promoting glucose utilization in skeletal muscle 14. It also regulates the hypothalamic-pituitary-adrenal axis to reduce stress hormones like cortisol 15, 16, thereby decreasing visceral fat breakdown and FFAs influx to the liver 15, 16.

Anaerobic exercise primarily involves high-intensity, short-duration activities, typically exceeding 60%-80% of an individual's maximal oxygen uptake (VO₂max) 17, 18. Energy is mainly derived from the phosphagen and glycolytic systems 17, 18, exemplified by resistance training and power training 14, 17, 19. Its potential hepatic effects include maintaining blood glucose stability by promoting gluconeogenesis, increasing hepatic glycogen storage in the long term 20, 21, and indirectly reducing hepatic lipid accumulation by improving insulin sensitivity 19, although its independent effects are generally weaker than those of aerobic exercise 14, 19.

High-intensity interval training (HIIT, intensity 80%-100% VO_2_max), sprint interval training (SIT, intensity > 100% VO_2_max), and moderate-intensity continuous training (MICT, intensity 40%-59% VO_2_max) are three common modalities 22, 23. They can all reduce the risk of MASLD and lower liver enzyme levels through shared pathways such as improving insulin sensitivity and regulating lipid metabolism 20, 23-25. [Table 2]

## 3. Effects of Exercise on Liver Health and Molecular Mechanisms

### 3.1 Glucose and lipid metabolism

Exercise energy metabolism exhibits intensity dependence. According to the "crossover concept," low intensity (< 40% VO_2_max) primarily relies on lipid oxidation, which peaks at moderate intensity (45%-65% VO_2_max), while high intensity (> 65% VO_2_max) shifts to carbohydrate dominance 26. During the initial phase of exercise (10-15 minutes), skeletal muscle uptake causes a transient decrease in plasma fatty acids 27; subsequently, activation of the β-adrenergic system increases lipolysis efficiency threefold 28, and sympathetic nervous system excitation increases adipose tissue blood flow 2-7 times 29, 30, synergistically promoting lipid mobilization.

At the molecular level, AMPK plays a significant regulatory role: exercise training significantly reduces hepatic diacylglycerol levels, particularly when AMPK function is normal 31. AMPK inhibits fatty acid synthesis by phosphorylating acetyl-CoA carboxylase (ACC) 32 and promotes mitochondrial biogenesis to enhance β-oxidation capacity. AMPK-deficient mice exhibit hepatic triglyceride (TAG) accumulation post-exercise, confirming its necessity 31. This kinase also restricts glycerolipid synthesis by inhibiting the SREBP-1c/SCD-1 axis 31, 33. In glucose metabolism, AMPK promotes hepatic glycogen storage via a dual mechanism: upregulating UDP-glucose pyrophosphorylase 2 (UGP2) expression 34 and activating glycogen synthase by increasing glucose-6-phosphatase (G6Pase) levels 31. It is noteworthy that core evidence for AMPK's role primarily comes from gene knockout animal models. Human AMPK isoforms are diverse and subject to complex regulation by genetics, nutrition, and disease states, posing challenges for directly validating its central role in the human liver.

Within the nuclear receptor pathway, exercise bidirectionally regulates the PPAR family: it inhibits PPARγ-mediated lipogenesis by upregulating HNF1A/IRF6 and balancing PGC1α/RORα 35-38; while simultaneously activating the PPARα signaling axis to promote CPT-1/Acox-1 expression, enhancing fatty acid oxidation 38, 39. High-intensity interval training (HIIT) can coordinately regulate PPAR family members, promoting PPARα and its downstream targets CPT1α and ACOX1 expression while suppressing PPARγ-mediated lipogenesis 40. However, differences exist in expression patterns, ligand specificity, and physiological functions of PPAR isoforms between humans and rodents, necessitating caution when extrapolating animal model results to human clinical contexts 41-45.

In insulin signal transduction, exercise improves glucose metabolism through multiple targets. Exercise significantly enhances the hepatic IRS/PI3K/AKT pathway, specifically by reducing IRS1 Ser307 phosphorylation [46]and increasing AKT Ser473 phosphorylation 38. This improved signaling cascade effectively enhances insulin sensitivity and glucose tolerance 47. Additionally, exercise suppresses gluconeogenesis by downregulating phosphoenolpyruvate carboxykinase (PEPCK) and G6Pase expression, while increasing hepatic glycogen storage 38.

**Figure 1:**
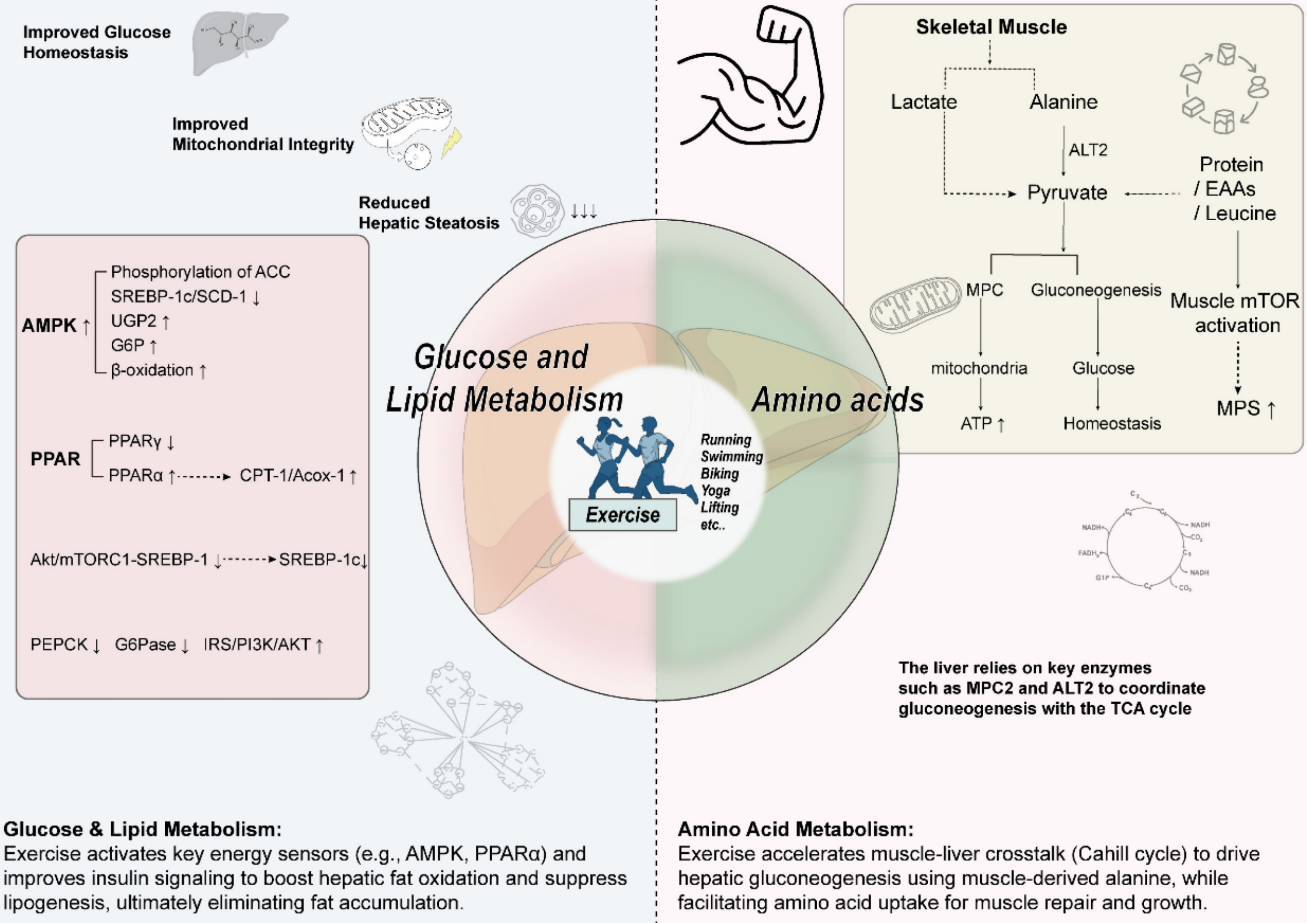
** Core regulatory role of exercise in hepatic energy metabolism**. In terms of glucose and lipid metabolism, exercise activates AMPK and PPARα, improves insulin signaling (IRS/PI3K/AKT), and suppresses the mTORC1-SREBP-1 axis. These coordinated actions enhance hepatic fatty acid oxidation, inhibit lipid synthesis, and thereby improve insulin sensitivity. Regarding amino acid metabolism, exercise accelerates muscle-liver crosstalk, driving hepatic gluconeogenesis with alanine as a key precursor. Concurrently, it enhances muscle protein synthesis and sensitivity to amino acids—particularly leucine—which synergistically promotes post-exercise anabolism and tissue repair.

The mTORC1 signaling axis also plays a significant role in exercise-mediated metabolic regulation. Overactivation of this pathway promotes lipid accumulation, whereas exercise intervention reduces hepatic triglyceride content by decreasing phosphorylation levels of the mTORC1 pathway marker S6K1 48. When exercise inhibits the Akt/mTORC1 - SREBP-1 pathway, it can reduce the expression and activity of SREBP-1, thereby diminishing the transcriptional activation of lipogenic genes like ACC and FAS, leading to decreased hepatic lipid synthesis and accumulation 49. [Fig 1]

### 3.2 Amino acids metabolism

Exercise significantly increases the body's demand for glucose to meet the energy requirements of muscle contraction 50. This process generates more pyruvate/lactate in skeletal muscle, with most lactate released into the bloodstream and taken up by the liver as a gluconeogenic substrate 51. Concurrently, alanine release from muscle increases and is transported via circulation to the liver 52. The liver processes alanine via deamination; its carbon skeleton is converted to pyruvate, which is then used for glucose production in the gluconeogenic pathway mediated by the Cahill cycle 52, 53. Exercise accelerates this cycle, promoting the efficient transport of alanine to the liver for gluconeogenesis to meet the body's glucose demand 53. The enhancement of hepatic gluconeogenesis relies on metabolic cross-talk between muscle and liver 51.

Research has identified key enzymes in this metabolic coordination: the mitochondrial pyruvate carrier (MPC) mediates pyruvate entry into mitochondria 54, 55, while alanine aminotransferase (ALT) catalyzes the conversion of alanine to pyruvate. Inhibiting hepatic MPC (particularly MPC2) and ALT2 activity impairs amino acid metabolism and gluconeogenic function. Liver-specific double knockout mice exhibit reduced gluconeogenic efficiency, decreased endogenous glucose production, impaired exercise endurance, and abnormal blood glucose levels 51. This study leveraged the advantages of animal models to clarify the necessity of MPC and ALT in metabolic coordination during exercise. However, complete loss-of-function mutations in such genes are rare in humans, and human glucose homeostasis regulation is more redundant and compensatory (e.g., renal gluconeogenesis). The severe phenotypes in knockout models may overestimate the absolute importance of single targets in human physiology. Research indicates that this inhibition also leads to a decline in tricarboxylic acid (TCA) cycle flux, primarily through the following mechanisms: (1) Insufficient substrate supply: MPC inhibition hinders pyruvate entry into mitochondria for conversion to acetyl-CoA (a key TCA cycle substrate); ALT2 inhibition reduces the conversion of alanine to pyruvate, collectively weakening TCA cycle substrate input 51. (2) Impaired metabolic flux: Reduced gluconeogenic efficiency disrupts the metabolism of shared intermediates (e.g., pyruvate, oxaloacetate) with the TCA cycle; concurrently, fluxes through anaplerotic reactions (replenishing intermediates) and cataplerotic reactions (removing intermediates) decrease, disrupting TCA cycle intermediate homeostasis 51. (3) Systemic metabolic dysregulation: Inhibition of MPC and ALT2 disrupts the coordination between hepatic gluconeogenesis, the TCA cycle, and inter-organ metabolic signaling (e.g., Cori cycle and Cahill cycle), ultimately compromising TCA cycle function 51.

Beyond regulating glucose metabolism, acute exercise (particularly resistance training) effectively stimulates muscle protein synthesis (MPS) 56. Protein supplementation around exercise synergizes with this stimulation to promote MPS 56. Studies confirm that acute amino acid intake significantly increases MPS rates 57. In the fasted state, muscle protein balance is often negative after a single bout of resistance exercise 58, but can become positive when combined with protein intake 59-61. Muscle sensitivity to amino acids is significantly enhanced post-exercise (lasting about 24 hours) 62. Supplementing protein during this period, compared to non-exercise periods, more efficiently provides a net stimulus for MPS and promotes protein accretion 28. Data show that immediate post-exercise protein intake can peak MPS rates within 3 hours and maintain elevated levels for 24 to 72 hours 62, 63. Therefore, timely and regular (every 3-4 hours) protein intake post-exercise maximizes its positive impact on muscle protein metabolism.

Furthermore, essential amino acids (EAAs), particularly leucine among the branched-chain amino acids (BCAAs), are crucial for regulating protein metabolism 56. Oral BCAAs rapidly increase plasma concentrations, providing key substrates for skeletal muscle MPS 56. Leucine intake can prolong the duration of protein synthesis 64-66, with its stimulatory effect being dose-dependent: plateauing at about 2g at rest 60, 64, but requiring about 3.5g after 60 minutes of moderate-intensity cycling 66. However, the regulation of MPS rate duration depends not only on leucine concentration but also on ATP status and the availability of other EAAs. Therefore, balanced intake of all EAAs is most effective for sustaining elevated MPS rates 67. [Fig 1]

### 3.3 Injury and repair

Exercise exerts protective effects against metabolic-associated liver injury through synergistic multi-mechanisms, with both common and specific pathways involved in models of diabetes and fatty liver disease.

For diabetes-associated liver injury, mechanisms primarily include metabolic regulation, oxidative stress alleviation, apoptosis inhibition, and inflammation modulation. Twelve weeks of aerobic exercise significantly improved metabolic disorders in db/db mice, manifested as reduced body weight and fasting blood glucose, and enhanced insulin sensitivity. Phenotypic validation confirmed that exercise ameliorated hepatic fat accumulation and functional injury 68. Regarding oxidative stress regulation, exercise can activate the Nrf2/ARE pathway, upregulating Nrf2 expression to promote SOD activity and reduce oxidative stress markers like MDA, exerting a protective effect 68-70. db/db mice are a classic model for studying type 2 diabetes, but their pathology progresses rapidly and severely, with liver injury often accompanied by extreme metabolic disturbances. The significant exercise effects observed in these genetically deficient animals may be less prominent in human diabetic patients, who typically have milder and slower-progressing conditions. Additionally, differences may exist in the target gene repertoire and activation thresholds of the Nrf2 pathway between rodents and humans. The anti-apoptotic effect of exercise on hepatocytes is mainly reflected in downregulating the phosphorylation levels of signaling proteins like p-JAK2, p-STAT3, reducing Bax/cleaved caspase-3 expression, and decreasing TUNEL-positive cell numbers, thereby inhibiting apoptosis pathway activation 68. At the inflammation level, exercise not only inhibits NF-κB pathway activation and reduces p-P65 expression but also modulates the concentrations of inflammatory mediators like IL-6, TNF-α and macrophage infiltration, forming a multi-dimensional anti-inflammatory effect 68.

**Figure 2:**
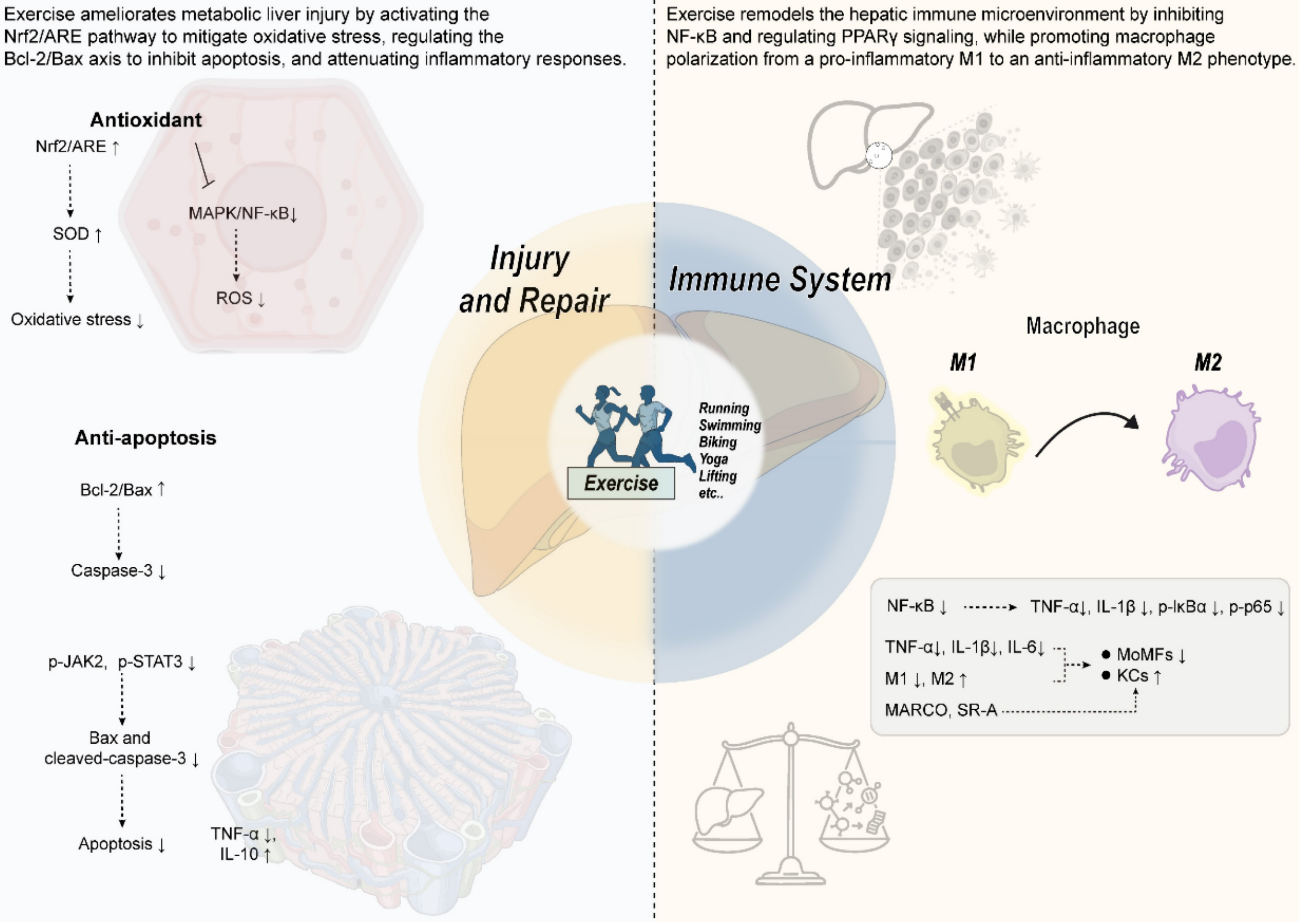
** Mechanisms by which exercise improves metabolic liver injury and the immune microenvironment.** Exercise exerts protective effects against metabolic liver injury and remodels the hepatic immune microenvironment through multi-pathway synergism. The key mechanisms include: (1) Antioxidant effects: Activation of the Nrf2/ARE pathway upregulates antioxidant enzymes such as SOD, reducing oxidative stress and ROS levels. (2) Anti-apoptotic and reparative effects: Upregulation of the Bcl-2/Bax ratio, inhibition of caspase-3 activity, and downregulation of signaling pathways such as p-JAK2/p-STAT3 collectively suppress hepatocyte apoptosis. (3) Immunomodulation and anti-inflammatory effects: Inhibition of the NF-κB pathway (reducing p-IκBα and p-p65) decreases the production of pro-inflammatory cytokines such as TNF-α and IL-1β. Meanwhile, exercise modulates macrophage polarization, promoting a shift from the pro-inflammatory M1 phenotype (CD86⁺) to the anti-inflammatory M2 phenotype (CD163⁺, CD206⁺), and enhances Kupffer cell function, thereby alleviating hepatic inflammation.

In MASLD models, exercise optimizes hepatic lipid homeostasis through bidirectional regulatory mechanisms: on one hand, promoting fatty acid β-oxidation by increasing ACC phosphorylation levels and cytochrome c content; on the other hand, inhibiting FAS expression to reduce lipid synthesis 71. Regarding cytoprotective mechanisms, exercise inhibits the mitochondrial apoptosis pathway by modulating the Bcl-2/Bax balance, reducing caspase-3 activity, thereby decreasing hepatocyte programmed cell death; and induces protective autophagy to promote lipid droplet degradation, which is particularly important for MASLD with impaired autophagy function 72-75. In oxidative stress regulation, exercise reduces ROS generation, enhances antioxidant enzyme expression, downregulates pro-inflammatory factors like TNF-α, and increases anti-inflammatory mediators like IL-10 via the MAPK/NF-κB pathway, thereby inhibiting macrophage infiltration and alleviating liver injury caused by MASLD 76, 77. However, most MASLD animal models are induced by high-fat diets or genetic modifications, developing significant steatosis and inflammation within weeks, which differs from the often years-long development of human MASLD. The rapid inhibition of apoptosis and induction of autophagy observed in short-term animal models may reflect an acute adaptive response to stress rather than long-term regulation of chronic human disease. Measuring hepatic autophagy flux in humans is extremely challenging. [Fig 2]

### 3.4 Immune system

Exercise significantly modulates the hepatic immune microenvironment, effectively attenuating liver inflammation through multi-target regulation of the NF-κB and PPARγ signaling pathways. Studies have found that long-term high-fat diet significantly activates the NF-κB signaling pathway, manifested as increased hepatic p-IκBα and p-p65 protein levels, and markedly elevated expression of pro-inflammatory factors TNF-α and IL-1β in serum and liver tissue 38. Swimming exercise can significantly suppress NF-κB pathway activation and reduce the levels of these inflammatory markers 38. In terms of PPARγ signaling regulation, exercise intervention works through multiple mechanisms: directly downregulating PPARγ and its downstream target genes (e.g., CD36, SCD1, PLIN2), reducing hepatic fatty acid uptake and lipid droplet accumulation 38; upregulating transcriptional repressors like HNF1A and IRF6, which bind to the PPARγ promoter region to inhibit its transcriptional activity 38; modulating the dynamic balance between the coactivator PGC1α [78]and the negative regulator RORα 35, thereby suppressing PPARγ transcriptional activity. The synergistic action of these molecular mechanisms ultimately leads to reduced lipogenesis and attenuated inflammatory response, improving hepatic metabolic homeostasis.

In the development of MASLD, the accumulation and activation of pro-inflammatory monocytes (MoMFs) play a key role in hepatic inflammation 79, 80. Exercise intervention significantly reduces the risk of progression to metabolic dysfunction-associated steatohepatitis (MASH) by modulating the migration and activation of MoMFs. Specific mechanisms include: (1) lowering the expression levels of hepatic pro-inflammatory cytokines (e.g., TNF-α, IL-1β); (2) reducing pro-inflammatory monocyte infiltration; (3) alleviating hepatic oxidative stress 81, 82. These mechanisms collectively inhibit inflammation triggered by immune cells, protecting the liver from MASH damage 82-85.

Additionally, exercise enhances immune function and anti-inflammatory effects through multiple mechanisms: on one hand, upregulating the expression of CD68 and scavenger receptors (such as MARCO, SR-A) on Kupffer cells (KCs), enhancing their phagocytic and endotoxin clearance capacity, while increasing blood dehydroepiandrosterone and inhibiting NFκB-p65 phosphorylation to reduce inflammation; on the other hand, accelerating endotoxin clearance, lowering levels of pro-inflammatory factors like TNF-α and IL-6, further optimizing KCs function and modulating immune responses 86. Exercise also has significant immunostimulatory effects in cancer patients. Studies show that acute exercise can significantly increase circulating natural killer (NK) cell numbers 87 and may induce lasting functional changes in NK cells, thereby enhancing the body's immune defense against cancer 88. Simultaneously, γδ T cells exhibit enhanced expansion efficiency and phenotypic remodeling upon bisphosphonate antigen activation following exercise-induced stress stimulation, consequently amplifying their cytotoxic effects against various hematologic malignancies 89. Notably, as pivotal components of the CD4/CD8 double-negative T cell subpopulation, the antitumor activity of γδ T cells is closely associated with IL-15 signaling pathway: IL-15 can activate surface effector molecules including NKG2D, DNAM-1, and NKp30, while promoting intracellular expression of perforin and granzyme B, ultimately augmenting this subpopulation's tumor-targeted cytolytic capacity 90.

Research indicates that HIIT has significant anti-inflammatory effects, downregulating the gene expression of pro-inflammatory cytokines such as TNF-α, MCP-1, IL-6, and IL-1β, thereby alleviating liver inflammation 40. Moreover, HIIT modulates macrophage polarization by reducing the expression of total macrophage markers (F4/80, CD68) and M1 markers (CD86), while increasing M2 markers (CD163, CD206), thereby improving inflammation. Mechanistic studies suggest that HIIT may promote hepatic macrophage polarization towards the M2 phenotype via an RORα-dependent KLF4 pathway, exerting anti-inflammatory effects 91. It is important to note that research on macrophage polarization heavily relies on flow cytometry and specific markers (e.g., CD86, CD206), whose expression and function are not entirely consistent between rodent and human macrophages. The clear M1-to-M2 shift observed in animal models may appear more blurred and dynamic in the complex human liver immune microenvironment. Additionally, the comparability of HIIT protocols (e.g., intensity, interval duration) between animal and human studies is problematic, as animals often endure higher absolute exercise loads, potentially leading to stronger immune stress responses. [Fig 2]

Additionally, the mechanism by which exercise suppresses hepatic immune cell activation through the regulation of lipotoxic metabolites has garnered increasing attention. Research has demonstrated that exercise effectively reduces hepatic levels of lipotoxic metabolites, particularly FFAs and ceramides, thereby elevating the activation threshold of immune cells and curbing excessive inflammatory responses. The underlying mechanisms involve exercise-induced upregulation of PGC-1α, which enhances mitochondrial fatty acid β-oxidation and reduces FFAs accumulation in hepatocytes 92. Moreover, chronic exercise lowers levels of long-chain ceramides, such as C16:0 and C18:0, in both serum and liver tissue; these molecules are critical mediators of apoptosis and inflammation 93. Furthermore, exercise activates the AMPK/mTOR pathway to induce autophagy, facilitating the clearance of damaged mitochondria and surplus lipids, thereby alleviating lipotoxic burden 92.

The reduction in these lipotoxic metabolites profoundly influences the hepatic immune microenvironment. On one hand, ceramides act as activating factors for the NLRP3 inflammasome; by downregulating ceramide levels, exercise suppresses NLRP3 activation, reduces the release of IL-1β and IL-18, and elevates the threshold for immune cell response to damage signals 93. On the other hand, exercise remodels the composition of hepatic immune cells, diminishing the infiltration of pro-inflammatory CD4+, T cells, B cells, and macrophages, thereby reducing susceptibility to acute injury 94. At the signaling pathway level, exercise enhances the interaction between macrophage migration inhibitory factor and CD74, inhibiting the activation of the lipotoxic stress-associated kinase JNK 95. Concurrently, it upregulates anti-apoptotic proteins, counteracting TNF-α-mediated inflammatory damage and reinforcing immune homeostasis 94. In summary, by systematically reducing levels of lipotoxic metabolites, exercise synergistically suppresses excessive hepatic immune activation at the levels of metabolic substrates, immune cell composition, and signaling pathways, thereby exerting a protective effect.

### 3.5 Myokines, sarcopenia and the muscle-liver axis

Exercise-derived myokines exert hepatoprotective effects through synergistic multi-pathways 96-98. Long-term regular exercise reduces myostatin levels, promoting intrahepatic fatty acid oxidation and alleviating fibrosis via the SMAD-AMPK/G6PD signaling axis 96-98. Acute exercise induces an increase in interleukin-6 (IL-6), improving iron metabolism via the IL-6/JAK2/STAT3 pathway and potentially attenuating ferroptosis by inhibiting tumor necrosis factor-α (TNF-α) activity 99-101. Endurance training promotes irisin secretion, activating the AMPK/p38 MAPK pathway and inhibiting the NLRP3 inflammasome, thereby reducing lipid peroxidation and inflammation 102-105. Exercise also upregulates FGF21, enhancing antioxidant capacity via the Nrf2/HO-1/GPX4 pathway 106-108. In the elderly, insulin-like growth factor 1 (IGF-1) elevated by resistance exercise alleviates oxidative damage via the IGF-1/mTOR pathway 109, 110. In summary, various myokines synergistically improve hepatic lipid metabolism and fibrosis by enhancing antioxidant defense (e.g., activating Nrf2/GPX4 101, 106), promoting fatty acid breakdown, inhibiting inflammation, and cell death. Different forms of exercise differentially affect myokine release, with resistance training potentially being more effective than aerobic exercise 4, 111, while combined endurance training may further optimize mitochondrial function and lipid metabolism, comprehensively enhancing liver protection 112.

The chemokine CX3CL1 plays a key role in regulating skeletal muscle metabolic adaptation and repair. In skeletal muscle, CX3CL1 primarily functions in an autocrine manner, driving the transition of fast-twitch fibers from the fast-twitch oxidative-glycolytic to purely glycolytic (type IIB), thereby enhancing anaerobic energy supply capacity; this transition is mainly mediated by modulating mitochondrial activity and myosin ATPase, with minimal impact on slow-twitch fibers 113, 114. Exercise training upregulates skeletal muscle CX3CL1 expression, a mechanism potentially related to Ca²⁺/CaMKII signal activation, which subsequently promotes glucose uptake and mitochondrial biogenesis, regulating muscle metabolism 114-116. Furthermore, the CX3CL1/CX3CR1 signaling axis is crucial in muscle injury repair, including promoting angiogenesis, mediating macrophage clearance of necrotic tissue, and coordinating muscle satellite cells with immune cells to accelerate regeneration 117, 118. It is worth noting that CX3CL1 not only exerts autocrine effects locally in skeletal muscle, but recent studies have revealed that it can also act on the liver and participate in regulating immune cell functions, suggesting that CX3CL1 may play an important role in muscle-liver crosstalk 119.

**Figure 3:**
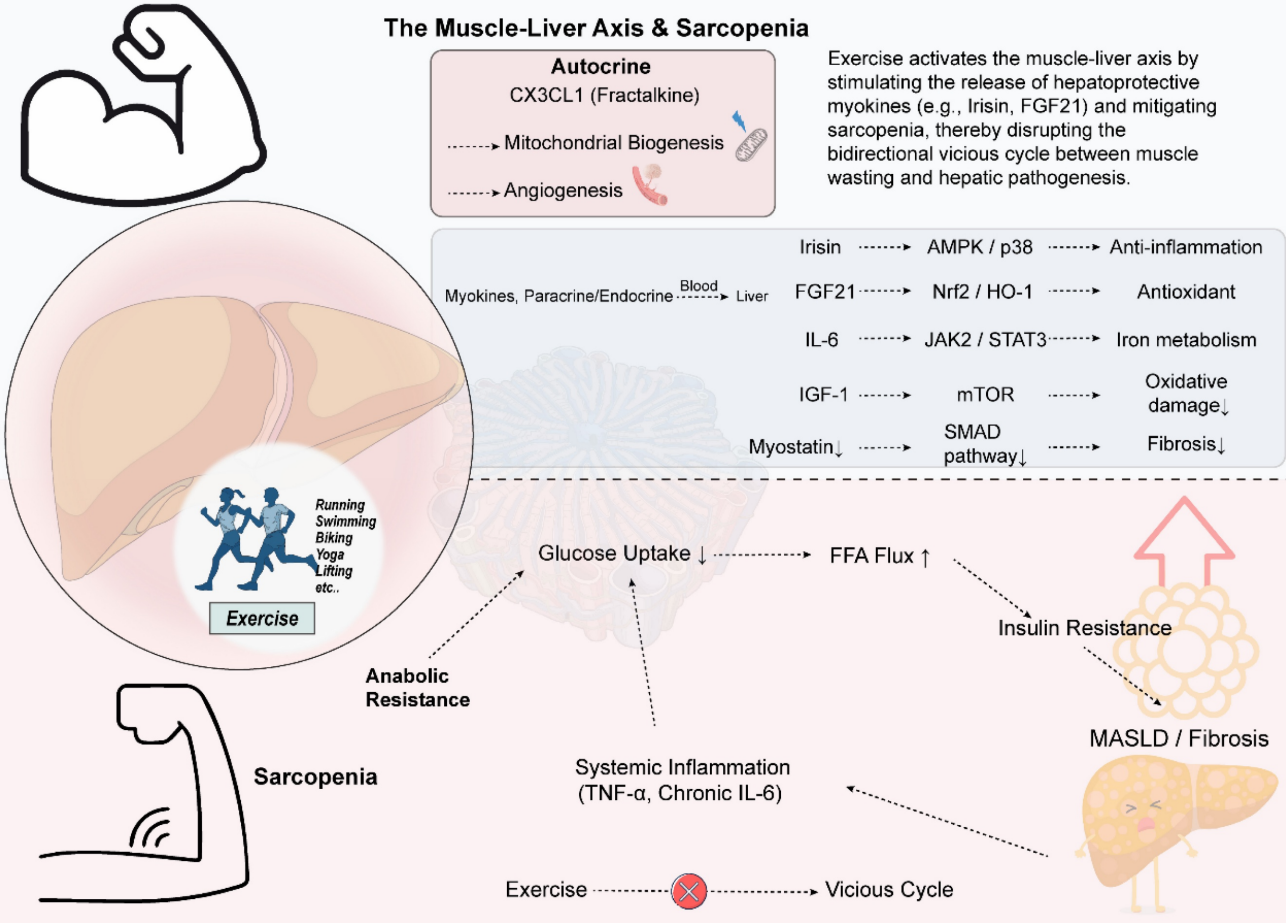
** Exercise modulates the muscle-liver axis to interrupt the vicious cycle between sarcopenia and liver disease.** Exercise stimulates the release of various myokines, such as Irisin, FGF21, and IGF-1, from skeletal muscle. These myokines act on the liver through the systemic circulation and exert protective effects via distinct molecular pathways: Irisin activates the AMPK/p38 pathway to reduce inflammation, FGF21 enhances the Nrf2-mediated antioxidant defense system, and IGF-1 alleviates oxidative damage through the mTOR pathway. Collectively, these mechanisms improve hepatic lipid metabolism and suppress fibrosis. Moreover, exercise upregulates the chemokine CX3CL1, which promotes mitochondrial biogenesis and angiogenesis within muscle tissue. Conversely, sarcopenia is characterized by anabolic resistance, reduced muscle glucose uptake, and systemic inflammation—marked by elevated levels of TNF-α and chronic IL-6. These alterations exacerbate insulin resistance and increase the influx of free fatty acids into the liver, thereby promoting hepatic steatosis and injury, and perpetuating a vicious cycle.Various forms of exercise can effectively target this axis, offering a therapeutic strategy to break the reciprocal worsening of sarcopenia and liver disease.

Sarcopenia, an age-related progressive loss of skeletal muscle mass and function, is closely associated with MASLD. The prevalence of sarcopenia in MASLD patients increases with the progression of liver fibrosis 120-122. The two conditions form a bidirectional vicious cycle via the "muscle-liver axis," with core pathological mechanisms including: skeletal muscle anabolic resistance, i.e., reduced responsiveness to nutritional and hormonal signals, which can be exacerbated by MASLD-related inflammation and metabolic disturbances 123-125; decreased muscle glucose uptake leading to insulin resistance, causing ectopic deposition of free fatty acids in the liver and promoting steatosis, while muscle loss further worsens systemic insulin resistance 123, 126; impaired metabolic flexibility in MASLD/MASH patients, manifesting as difficulty switching between fatty acid oxidation and glucose utilization, thereby worsening intrahepatic lipid accumulation and insulin resistance 127; systemic inflammation and signaling dysregulation, where factors like TNF-α and IL-6 promote both muscle catabolism and liver fibrosis, coupled with imbalances in myokine and hepatokine secretion and factors like vitamin D deficiency, collectively constituting this complex pathological network 128-130. Therefore, comprehensive interventions targeting the "muscle-liver axis" (e.g., nutritional and exercise therapies) can provide multi-target strategies for MASLD/MASH by modulating these mechanisms 123. [Fig 3]

## 4. The Specific Effects of Exercise on Different Liver Diseases

### 4.1 Exercise and MASLD

MASLD is a liver disease caused by multi-system metabolic imbalances. Its diagnosis requires hepatic steatosis ≥ 5% accompanied by conditions such as overweight/obesity, type 2 diabetes, or metabolic dysregulation, while excluding other liver diseases and alcohol abuse 131. The disease is categorized into simple steatosis and MASH, with the latter carrying a higher risk of progression to cirrhosis and HCC due to accompanying inflammation and fibrosis 132. Epidemiological data show a global MASLD prevalence exceeding 30%, with significant regional variations: highest in South America (> 40%), lowest in Africa (14%), and fastest growth in the Middle East and North Africa 133, 134. Incidence is particularly high among males and obese populations, while weight loss of 5%-10% can effectively reverse hepatic steatosis and fibrosis, highlighting the necessity of preventive interventions for high-risk groups 135.

Lifestyle interventions centered on weight loss and regular exercise are foundational treatment strategies 136. Aerobic exercise improves liver fat content through dual mechanisms: on one hand, enhancing cardiorespiratory function, promoting hepatocyte and skeletal muscle mitochondrial proliferation and functional optimization, thereby increasing fatty acid oxidation efficiency 137; on the other hand, modulating hormone balance, such as stimulating growth hormone secretion to accelerate visceral fat breakdown and reduce fatty acid transport to the liver via the portal system 138. Exercise also enhances insulin sensitivity, inhibiting lipolysis and FFAs release 139, 140. Common aerobic exercise forms include walking, treadmill, and stationary cycling, which offer controllable intensity and strong site adaptability. Compound exercises like rowing machines and ellipticals are increasingly popular due to their ability to activate multiple muscle groups. Although high-intensity aerobic training is less commonly applied, studies confirm that performing it three times weekly (for 8 weeks) or five times weekly (for 6 months) can reduce liver fat by 2.4% and 5.0% respectively, suggesting its potential value 141, 142.

Research on exercise dosage shows that 150-240 minutes of moderate-intensity aerobic exercise per week can reduce hepatic fat by 2%-4%, and even 135 minutes still yields significant effects 4, 142. This approach improves central obesity and cardiopulmonary function even without weight loss, demonstrating clinical feasibility. Resistance training, as an important complement to aerobic exercise, although less stimulating to the cardiopulmonary system, effectively enhances muscle mass and metabolic regulation capacity 143, 144. Population studies have confirmed a negative correlation between resistance training and hepatic fat levels, with high-intensity resistance training showing superior effects 145, 146. An 8-week intervention can reduce hepatic steatosis by 13% independent of weight change 147 but due to study heterogeneity, its exact efficacy still needs to be verified 146. Currently, there is a lack of direct mechanistic research on the effects of resistance training on intrahepatic lipids and metabolism 143, 148. HIIT, which alternates high-intensity exercise (reaching 80%-100%HRmax) with rest, shows lipid-lowering effects comparable to MICT 137, 149. However, due to limitations in sample size and study dimensions, more evidence is needed to compare their efficacy 24, 149. MICT, as a traditional model, requires maintaining 45%-80%HRmax for 20-60 minutes 24, 150. Studies have confirmed that its regular implementation (≥ 5 times per week) can reduce MASLD incidence by 16% and increase remission rates by 40%, with exercise volume accumulation positively correlated with disease improvement 151.

**Figure 4:**
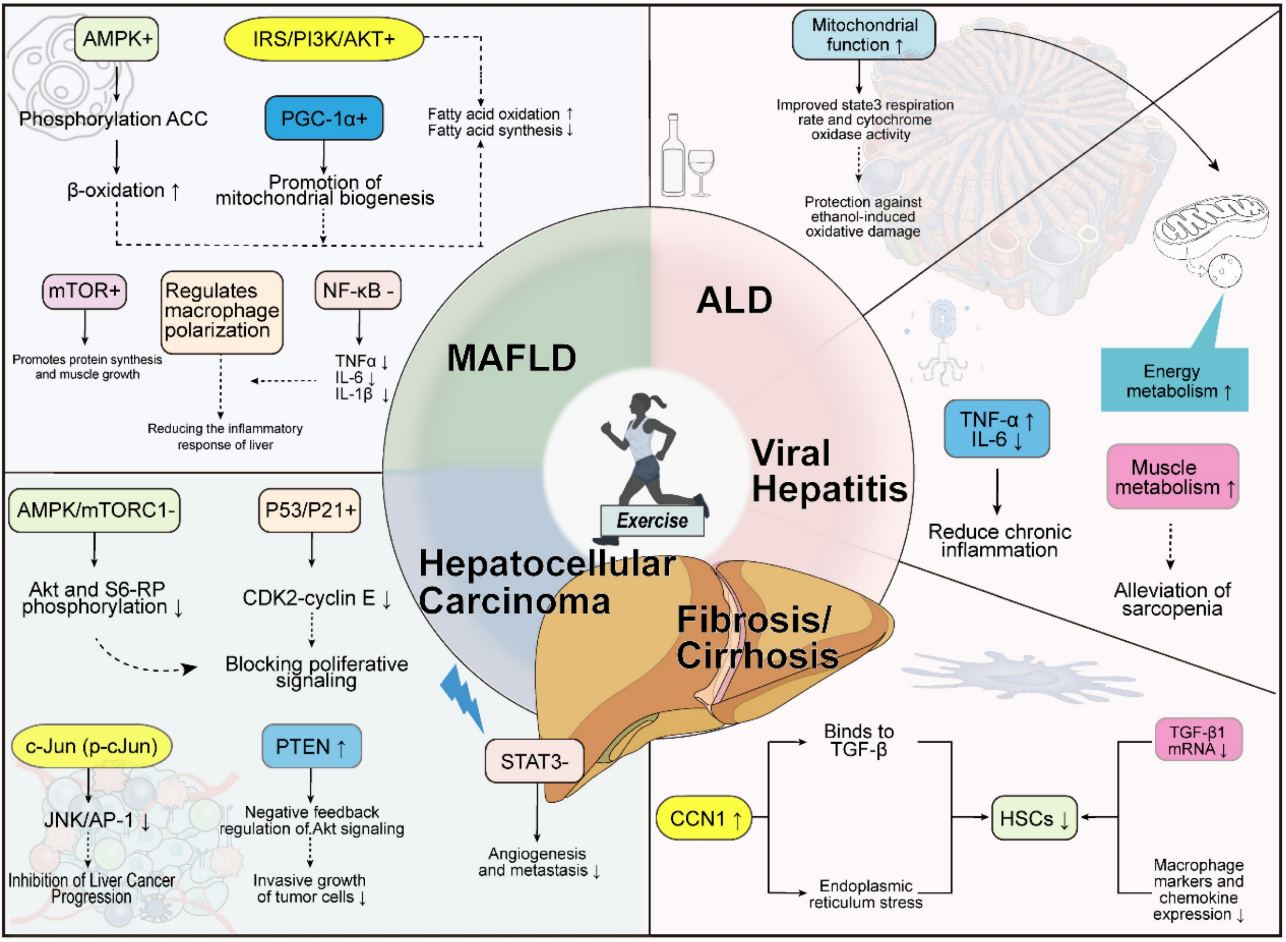
** Specific Effects of Exercise on Different Types of Liver Diseases**. In MASLD, exercise activates the AMPK pathway to promote ACC phosphorylation and enhance beta-oxidation. It also facilitates mitochondrial biogenesis via the IRS/PI3K/AKT pathway and PGC-1α. Furthermore, exercise modulates macrophage polarization through mTOR and reduces levels of NF-κB-related inflammatory factors, thereby alleviating hepatic inflammation. In ALD, exercise improves mitochondrial function, increases the tricarboxylic acid (TCA) cycle rate and cytochrome oxidase activity, and protects the liver from ethanol-induced oxidative damage. For viral hepatitis, exercise regulates inflammatory factors such as TNF-α and IL-6, mitigating chronic inflammation. It also enhances muscle metabolism, which may help alleviate sarcopenia. Regarding fibrosis/cirrhosis, exercise upregulates CCN1, which binds to TGF-β and induces endoplasmic reticulum stress, thereby reducing HSCs. It also decreases TGF-β1 mRNA levels and suppresses the expression of macrophage markers and chemokines. In HCC, exercise inhibits Akt and S6-RP phosphorylation via the AMPK/mTORC1 pathway and blocks cell proliferation signals through the P53/P21 pathway. It also suppresses the JNK/AP-1 pathway via phosphorylated c-Jun (p-c-Jun), thereby impeding HCC progression. Additionally, exercise negatively regulates Akt signaling through PTEN, reducing invasive tumor growth, and inhibits the STAT3 pathway, thereby diminishing angiogenesis and metastasis.

In summary, the comprehensive application of aerobic exercise, resistance training, and high-intensity interval training holds significant value for the prevention and treatment of MASLD. Regular moderate-to-high-intensity physical exercise not only reduces the risk of disease onset but also promotes disease remission, providing evidence-based support for clinical practice. [Fig 4]

### 4.2 Exercise and ALD

ALD is a group of liver disorders caused by chronic excessive alcohol consumption. Its clinical manifestations and histopathological features include alcohol-associated steatohepatitis (ASH), cirrhosis, and HCC 152. Among individuals with long-term heavy alcohol consumption, over 90% develop alcohol-associated fatty liver, characterized by abnormal fat accumulation in hepatocytes 152. The mechanisms underlying steatosis are complex, involving impaired mitochondrial fatty acid β-oxidation, abnormal lipid transport from peripheral tissues to the liver, and dysregulation of lipid metabolism-related transcription factors 152.

An 8-week animal study explored the interactive effects of exercise and ethanol on hepatic mitochondrial function in female Sprague-Dawley rats. Results showed that ethanol exposure specifically impaired mitochondrial function: state 3 respiratory rate (with glutamate as substrate) and cytochrome oxidase activity significantly decreased, with the most pronounced decline in the sedentary ethanol (S/E) group compared to others (p < 0.05). Notably, although ethanol intervention reduced maximal oxygen consumption rate, indicators reflecting oxidative phosphorylation coupling efficiency, such as respiratory control ratio (RCR) and phosphorus-to-oxygen ratio (P:O), showed no significant change, suggesting the damage primarily affects the electron transport chain rather than ATP synthesis coupling 153.

Regarding the effect of exercise intervention, daily 2-hour swimming training itself did not improve baseline mitochondrial function parameters (state 3 respiration, RCR, and P:O) but demonstrated significant protective effects under ethanol exposure. The swim-ethanol combined group maintained better mitochondrial oxidative function compared to the S/E group, with a 21.3%±3.2% increase in state 3 respiratory rate (p < 0.01) and cytochrome oxidase activity restored to 89.7%±5.4% of control levels. This confirms that endurance exercise can effectively counteract ethanol-induced oxidative damage by enhancing mitochondrial functional adaptability 153. These findings reveal a unique protective mechanism of exercise intervention in alcohol-associated liver injury: regular endurance training, while not directly enhancing baseline mitochondrial function, improves mitochondrial stress tolerance, maintaining functional homeostasis under pathological conditions. It is noteworthy that the study used female rats, while significant gender differences exist in human ALD (higher prevalence in males, but faster progression in females) 154. Sex hormones have important effects on liver metabolism and exercise responses 155, 156, thus gender factors must be considered when inferring results 157, 158. Furthermore, in animal experiments, ethanol administration is a controlled, forced intake, differing from the complex and variable drinking patterns in humans. The role of exercise in preventing (rather than treating) established alcohol-associated hepatitis or cirrhosis still requires more human prospective studies for confirmation. [Fig 4]

Furthermore, when discussing the protective effects of exercise, it is essential to acknowledge the dual nature of oxidative stress. While regular moderate exercise can induce adaptive protective effects by upregulating the endogenous antioxidant system 159, acute, high-intensity, or unaccustomed exercise may trigger a transient burst of ROS, thereby exacerbating tissue damage 159. Studies have shown that even a single bout of acute exercise in heavy drinkers can further deplete their already low GSH levels, accompanied by a significant increase in oxidative damage markers such as thiobarbituric acid reactive substances, indicating a severe deficiency in antioxidant reserves under acute stress 160.

For patients with severe alcoholic hepatitis, whose livers are already in a state of extreme oxidative stress and intense inflammation, the timing and intensity of exercise interventions require careful consideration. During the acute phase of the disease, treatment should primarily focus on bed rest and nutritional support, avoiding any high-intensity physical activity that could lead to a surge in ROS and potentially exacerbate hepatocyte apoptosis and necrosis 161. Once the patient's condition stabilizes and they enter the rehabilitation phase, exercise prescriptions should adhere to the principle of "low-intensity initiation and gradual progression" 162. Current clinical consensus suggests that patients with advanced liver disease can engage in low-to-moderate intensity aerobic training, supplemented by light resistance exercises, with close monitoring of liver function throughout the process 163. This approach aims to harness the long-term antioxidant benefits of exercise while effectively avoiding the potential risks associated with acute increases in oxidative load.

### 4.3 Exercise and viral hepatitis

Acute hepatitis is a common liver disease with diverse but regular clinical manifestations. Its early symptoms mainly include gastrointestinal symptoms such as anorexia, malaise, nausea, and myalgia, typically occurring 1-2 weeks before the onset of jaundice 164.

Regarding rest and exercise for acute hepatitis patients, traditional views advocate strict bed rest to avoid physical exertion. Clinical observations have found that vigorous exercise during the incubation period may lead to rapid deterioration of the condition, even endangering life 165, 166. To explore the impact of exercise on the course of acute viral hepatitis, researchers conducted a controlled study. Twenty-five hospitalized patients were randomly assigned to an exercise group and a control group. The exercise group performed moderate-intensity exercise twice daily (reaching 70% of maximal exercise intensity), while the control group adhered to strict bed rest. Results showed no significant difference between the two groups in the recovery time of biochemical markers or histopathological changes 167. This finding provides new insights into rehabilitation strategies for acute hepatitis patients.

In the field of chronic liver disease (CLD), the diagnosis is made when liver inflammation and/or necrosis persist for more than 6 months 164. CLD is a significant cause of premature death in the working-age population 168, 169. This disorder induces a plurality of pathophysiological alterations in skeletal muscle, encompassing metabolic, cellular, vascular, and inflammatory changes, which culminate in sarcopenia 170. These changes, combined with malnutrition and physical inactivity 171, contribute to a high prevalence of frailty (40%-70%) secrete fibrogenic factors and inflammatory mediators 172. Although theoretically exercise might have positive effects on chronic hepatitis, studies show only a neutral impact on chronic persistent hepatitis 173. To assess the effect of exercise intervention on chronic hepatitis, researchers implemented a 12-week standardized interval training program for 17 patients with chronic active hepatitis, monitoring liver function tests, maximal oxygen uptake, and exercise tolerance. Results showed stable serum transaminase levels during training, while maximal oxygen consumption and exercise tolerance significantly improved 174. Therefore, patients with chronic active hepatitis can not only tolerate regular exercise but may also benefit from its effects on liver health. However, this study had a relatively small sample size, and the historical classification "chronic active hepatitis" has now been replaced by more specific etiological and pathological staging systems. Physiological and pathological responses to exercise may differ among chronic liver diseases of various etiologies. [Fig 4]

### 4.4 Exercise and liver fibrosis, cirrhosis

Liver fibrosis is a common dynamic pathological process in various chronic liver injuries, characterized by excessive extracellular matrix accumulation and reduced degradation by metalloproteinases 175. Activated hepatic stellate cells (HSCs) play a critical role in this process, serving as the primary source of extracellular matrix 176 and transforming into myofibroblasts 177. Although multiple etiologies of chronic liver disease can directly activate HSCs, the core driver of sustained fibrosis progression is the inflammatory microenvironment.

Studies indicate that intracellular lipid accumulation in hepatocytes promotes the transition from steatosis to MASH 178, 179. Lipotoxicity causes hepatocyte damage, triggering cellular stress, apoptosis, and death 180, 181. HSCs and Kupffer cells, upon uptake of apoptotic bodies released by hepatocytes, secrete fibrogenic factors and inflammatory mediators 182. Substances secreted by Kupffer cells attract immune cell aggregation, exacerbating inflammation, activating HSCs, and inducing metabolic-associated liver fibrosis 183.

Exercise offers multiple benefits for liver fibrosis. Studies show that exercise increases the expression of CCN1, a senescence marker in HSCs 85. CCN1 can bind to TGF-β, inhibiting SMAD signaling and reducing collagen deposition 184. It is also associated with endoplasmic reticulum stress-induced HSC apoptosis 185, thereby reducing HSC activation and inhibiting fibrogenesis. Another study showed that 16-week of aerobic exercise intervention decreased hepatic TNF-α mRNA levels, alleviated inflammation, suppressed TGF-β1 mRNA expression, and reduced collagen deposition and macrophage markers and chemokine expression in the liver 83. It is noteworthy that animal models used to induce liver fibrosis are based on acute or subacute injury, whose pathophysiology fundamentally differs from the slow, multi-year progression of fibrosis in human chronic liver disease. The anti-fibrotic effects demonstrated by exercise in acute injury models are not equivalent to its ability to reverse established fibrosis or even cirrhosis in human livers. Human studies primarily rely on non-invasive indicators like liver elastography to indirectly assess fibrosis changes, which differs from the molecular analysis of HSC phenotype and extracellular matrix components possible in animal experiments.

Cirrhosis is a global disease with high morbidity and mortality, characterized by the replacement of healthy liver parenchyma with fibrous tissue and regenerative nodules, leading to portal hypertension and liver failure 183, 186. Treatment focuses on controlling the etiology and managing complications, with liver transplantation as a last resort 187-189. In recent years, the role of exercise intervention in the treatment and rehabilitation of cirrhosis has gained increasing attention. Research confirms that regular aerobic exercise significantly improves physical function in cirrhotic patients. If patients engage in supervised aerobic exercise at least twice weekly, such as slow jogging on a treadmill or whole-body aerobic training using a bicycle ergometer, for 2-3 months, their peak oxygen uptake can increase by 1.7 to 5.3 mL/kg/min 190, 191. Clinically, doctors often recommend that patients with compensated cirrhosis develop and implement a progressive exercise plan. Various aerobic exercises are effective and safe for this population. Walking is favored for its simplicity; swimming reduces body burden due to water buoyancy and minimizes joint stress; and spinning effectively enhances cardiorespiratory function and is also popular 173, 192. To ensure safety and effectiveness, patients are advised to start with low-load training, initially choosing short-duration, low-intensity activities, and gradually increasing duration, intensity, and difficulty as the body adapts. This exercise intervention strategy helps improve physical function and quality of life. [Fig 4]

### 4.5 Exercise and liver cancer

Early clinical symptoms of HCC are often insidious, with approximately 70% of patients losing the opportunity for curative treatment at diagnosis. The high five-year postoperative recurrence rate severely impacts patients' physiological function and mental health 193.

Exercise intervention inhibits the occurrence and progression of HCC through a multidimensional molecular regulatory network, involving cell cycle regulation, signaling pathway inhibition, and tumor microenvironment remodeling. In the molecular regulatory network, the AMPK/mTORC1 signaling axis plays a central role: exercise-induced AMPK phosphorylation disrupts mTOR complex stability, inhibits mTORC1 activity, and blocks cell proliferation signals 194. This process is accompanied by reduced phosphorylation levels of key downstream effectors Akt and S6-RP, with Akt inhibition forming a cascade amplification effect as an upstream activator of mTORC1. Simultaneously, STAT3 signaling inhibition significantly weakens tumor angiogenesis and metastatic potential 195.

In cell cycle regulation, exercise induces cell cycle arrest by activating the p53/p27 pathway. Experiments confirm that exercise significantly increases hepatic p53 Ser15 phosphorylation; with activated p53 upregulating the cell cycle inhibitor p27, specifically inhibiting CDK2-cyclin E complex function, thereby curbing abnormal hepatocyte proliferation. Additionally, exercise exerts anti-cancer effects by inhibiting the JNK1 signaling pathway: reducing phosphorylated c-Jun (p-cJun) accumulation and blocking JNK/AP-1 signaling axis activation, which is closely associated with MASH and HCC progression 196.

The tumor suppressor PTEN plays a key regulatory role in exercise intervention. Exercise upregulates PTEN expression, forming a negative feedback loop on Akt signaling pathway, effectively inhibiting invasive tumor growth. Phenotypically, exercise significantly reduces the proportion of active tumor areas, decreases CD31-positive vascular density, and suppresses tumor angiogenesis. Notably, exercise-induced metabolic improvements (e.g., enhanced insulin sensitivity, reduced hepatic lipid deposition) may indirectly inhibit HCC progression by reshaping the tumor microenvironment. However, the clear pathway inhibition observed in animal experiments may be invalidated or altered in human HCC due to factors like frequent TP53 mutations, PTEN loss, or Wnt/β-catenin pathway activation. Therefore, when developing exercise as an adjunctive therapy for human HCC, tumor molecular subtypes must be considered 194.

It is noteworthy that the relationship between exercise intensity and anti-tumor effects is nonlinear. Experimental data show that moderate-intensity training can significantly activate tumor-suppressive pathways, inducing tumor cell apoptosis more efficiently than low-intensity training (< 50% VO_2_max), while high-intensity training (> 80% VO_2_max) may exert pro-carcinogenic effects through mechanisms like oxidative stress 197, 198. This dose-dependent characteristic highlights the need for individualized exercise prescriptions in clinical practice. [Fig 4]

## 5. Potential Molecular Therapies

Exercise brings multiple clinical benefits to liver disease patients, including improved aerobic capacity, increased muscle mass, reduced fatigue, and enhanced quality of life 199. The systemic metabolic remodeling induced by exercise suggests that key molecular changes during this process could serve as potential drug targets for treating liver diseases. However, when translating these targets into clinical therapies, their respective limitations and safety issues must be fully considered.

AMPK, as a core energy-sensing molecule, is an important intervention target. Its direct activator, AICAR, has shown potential in preclinical studies to mimic exercise benefits 200. However, as an "exercise mimetic," its long-term safety, potential to interfere with exercise adaptation, and drug specificity (avoiding off-target effects) remain major challenges. Although the clinically common drug metformin can indirectly activate AMPK, its inhibition of mitochondrial complex I may attenuate exercise-induced metabolic adaptations. Furthermore, metformin is not yet approved for treating MASLD, and its liver-specific effects and long-term benefits require more evidence.

Thiazolidinediones (TZDs) improve insulin resistance and hepatic steatosis by activating PPARγ 201-203. However, their clinical application is limited by significant safety concerns, including weight gain, edema, increased risk of congestive heart failure, and fracture risk. Rosiglitazone has also been controversial due to potential cardiovascular risks. Although its hepatic benefits may stem from preferential effects on peripheral adipose tissue 204, 205, side effects associated with systemic PPARγ activation limit its widespread use in MASLD/MASH. Developing liver-specific PPARγ modulators or partial agonists may be a direction, but their efficacy-safety balance still needs validation.

Inhibitors targeting stearoyl-CoA desaturase 1 (SCD1), such as CAY10566, show potential in preclinical models for reducing hepatic steatosis and enhancing lipophagy 206. However, SCD1 plays important roles in lipid synthesis in various tissues (e.g., skin, pancreas). Systemic inhibition may lead to adverse effects like skin barrier dysfunction and pancreatitis. Developing liver-targeted SCD1 inhibitors is a key challenge in translational research.

Overactivation of the mTORC1 signaling pathway is closely associated with metabolic diseases 48. However, its inhibitors (e.g., rapamycin) face significant limitations in clinical application. mTORC1 is widely involved in systemic cell growth, proliferation, and immune regulation; inhibiting it can cause metabolic side effects like hyperglycemia, hyperlipidemia, and exacerbated insulin resistance, as well as issues like oral ulcers and immunosuppression 207, 208. Therefore, developing tissue-specific (especially liver-specific) mTOR modulation strategies or identifying more selective downstream effectors is key to improving the therapeutic window.

Evidence indicates that bioactive molecules capable of mimicking the effects of exercise may contain components beneficial to hepatic metabolism. For instance, SERCA activators can markedly reduce hepatic lipid accumulation and inflammatory responses by improving mitochondrial function and alleviating endoplasmic reticulum stress, thereby playing an important protective role in maintaining liver metabolic health 209. On the other hand, the mitochondrial DNA-derived peptide MOTS-c has been identified as a key signaling molecule mediating communication between mitochondria and the nucleus as well as other organs. As a potent exercise mimetic, MOTS-c can significantly enhance insulin sensitivity and modulate lipid metabolism. In models of liver disease, this peptide exerts anti-diabetic hepatic fibrotic effects by regulating the Keap1-Nrf2-Smad2/3 signaling pathway, offering a potential therapeutic strategy for liver injury induced by metabolic disorders 210.

In summary, although several highly promising molecular targets have been identified based on exercise biology mechanisms, their translation into clinical therapies still faces challenges related to specificity, safety, and uncertainty of long-term benefits 211. Future research needs to focus on developing tissue-specific delivery systems, exploring more precise downstream effector molecules, and advancing clinical translation based on solid preclinical safety assessments 212. Simultaneously, exercise itself, as a safe and multi-beneficial foundational intervention, warrants in-depth exploration of its synergistic effects with these potential targeted therapies 213.

## 6. Summary and Outlook

The liver, as a core organ of the human body, plays key roles in energy metabolism, protein synthesis, and toxin excretion. Liver injury can lead to issues like hypoglycemia and lipid metabolism disorders, severely impacting health. Common liver diseases include MASLD, ALD, various viral hepatitis, liver fibrosis, cirrhosis, and liver cancer. These diseases not only have high incidence rates and serious consequences but also possess unique pathogenesis, transmission routes, symptoms, and prognoses.

Compared to pharmacotherapy alone, exercise intervention demonstrates unique comprehensive advantages in liver disease treatment, offering multi-target. Exercise activates signaling pathways like AMPK/mTORC1 and p53/p27, improving insulin resistance, regulating lipid metabolism, and directly inhibiting abnormal hepatocyte proliferation and tumor angiogenesis. This multi-dimensional regulatory effect is difficult to achieve with a single drug. Furthermore, exercise intervention can synchronously improve systemic metabolic status, including weight reduction, amelioration of glucose and lipid metabolism disorders, and reduction of oxidative stress and inflammation, thereby creating a more favorable microenvironment for the liver. Additionally, exercise possesses a unique "exercise adaptation" effect, enhancing mitochondrial functional reserve and improving the liver's tolerance to various injurious factors—a form of protection that drugs cannot replicate. Moreover, exercise intervention avoids potential side effects and drug resistance issues associated with long-term medication and is more cost-effective. Most importantly, exercise improves patients' quality of life, achieving holistic enhancement of physical and mental health through overall modulation of the neuro-endocrine-immune network. These advantages make exercise intervention an indispensable component of liver disease management.

Future research can utilize multi-omics technologies to systematically analyze the mechanisms underlying exercise-induced hepatic protective adaptation, particularly the regulatory effects of exercise on the hepatic stellate cell-immune cell interaction network. Concurrently, developing artificial intelligence-based models to predict exercise efficacy, integrating biomarkers such as gut microbiota and circulating exosomes, will provide new paradigms for precision exercise medicine. It is noteworthy that while exercise mimetic drugs can partially replicate exercise effects, their potential interference with adaptive exercise responses requires careful evaluation. At the translational application level, there is an urgent need for large-scale clinical studies to validate the long-term benefits of different exercise modalities for patients with end-stage liver disease and to establish remote exercise monitoring systems based on wearable devices to improve treatment adherence.

Looking ahead, exercise intervention will transcend its traditional positioning as an adjunctive therapy. Through multi-dimensional integration with molecular targeted therapies, nutritional modulation, and psychological interventions, it will become a crucial pillar of comprehensive liver disease management. The development of this field requires not only deepening fundamental mechanistic research but also collaboration across multiple disciplines including clinical medicine, exercise science, and bioinformatics, ultimately achieving a shift from "population recommendations" to "individualized prescriptions" and providing innovative solutions for global liver health management.

## Author Contributions

Chen Cheng, Wentao Xu made substantial contributions to the conception and design of the work and drafted the manuscript; Shanshan Wu and Jia Gao critically revised the manuscript for important intellectual content; Li Zuo and Hua Wang finally approved the version to be published; Jianshang Huang and Yunsheng Dong and Jingjing Ji agreed to be accountable for all aspects of the work, ensuring that any issues related to the accuracy or integrity of any part of the work are appropriately investigated and resolved

## Figures and Tables

**Table 1:** Key metabolites, immune cells, and inflammatory mediators involved in liver diseases.

Liver Disease	Metabolites	Immune Cells	Inflammatory Mediators	Reference
MASLD	TAG	MoMFs	TNF-α	31,79,80,82-85
	ACC		IL-1β	32,71,82-85
	SCD-1			31, 33
	Acox-1			38, 39
	CPT-1			38, 39
ALD		Kupffer cells		86
Viral hepatitis		NK cells	TNF-α	68,86,87
			IL-6	86
Liver fibrosis/cirrhosis		Kupffer cells	TGF-β	182,183,184
			TNF-α	83
HCC		NK cells		88
		γδ T cells		90

**Table 2:** Classification of exercise intensities and their underlying mechanisms for improving liver function.

Exercise Category	Intensity Classification/Assessment Indicators	Specific Exercise Forms	Effects/Mechanisms on Liver Function	Reference
Aerobic Exercise	Moderate intensity, highly sustainable	Running, brisk walking, cycling, swimming	Enhances insulin sensitivity, inhibits hepatic gluconeogenesis, reduces hepatic free fatty acid deposition, promotes skeletal muscle glucose utilization, lowers cortisol levels, reduces visceral fat breakdown	12-16
Anaerobic Exercise	High intensity, short duration (> 60%-80% VO2max)	Resistance training, plyometric training	Promotes gluconeogenesis to maintain blood glucose, increases hepatic glycogen reserves, improves insulin sensitivity (indirectly reduces hepatic lipid accumulation)	14, 17-21
HIIT	80%-100% VO2max	Intermittent high-intensity exercise	Improves insulin sensitivity, regulates lipid metabolism, reduces MAFLD risk, improves liver enzyme levels	20, 22-25
SIT	> 100% VO2max	Extremely high-intensity sprints	Improves insulin sensitivity, regulates lipid metabolism, reduces MAFLD risk, improves liver enzyme levels	22-25
MICT	40%-59% VO2max	Continuous moderate-intensity exercise	Improves insulin sensitivity, regulates lipid metabolism, reduces MAFLD risk, improves liver enzyme levels	22-25

## Abbreviations

MASLD: metabolic dysfunction-associated steatotic liver disease
ALD: alcohol-related liver disease
ASH: alcohol-associated steatohepatitis
HCC: hepatocellular carcinoma
METs: metabolic equivalents
%HRmax: percentage of maximal heart rate
FFAs: free fatty acids
VO_2_max: maximal oxygen uptake
HIIT: high-intensity interval training
SIT: sprint interval training
MICT: moderate-intensity continuous training
MPC: mitochondrial pyruvate carrier
ALT: alanine aminotransferase
MPS: muscle protein synthesis
EAAs: essential amino acids
BCAAs: branched-chain amino acids
ATP: adenosine triphosphate
Nrf2: nuclear factor erythroid 2-related factor 2
ARE: antioxidant response element
SOD: superoxide dismutase
MDA: malondialdehyde
p-JAK2: phosphorylated Janus kinase 2
p-STAT3: phosphorylated signal transducer and activator of transcription 3
Bax: BCL2-associated X protein
NF-κB: nuclear factor kappa-light-chain-enhancer of activated B cells
IL-6: interleukin-6
TNF-α: tumor necrosis factor alpha
ACC: acetyl-CoA carboxylase
FAS: fatty acid synthase
Bcl-2: B-cell lymphoma 2
ROS: reactive oxygen species
IL-10: interleukin-10
MAPK: mitogen-activated protein kinase
TUNEL: terminal deoxynucleotidyl transferase dUTP nick end labeling
IL-1β: interleukin-1 beta
CD36: cluster of differentiation 36
SCD1: stearoyl-CoA desaturase 1
PLIN2: perilipin 2
HNF1A: hepatocyte nuclear factor 1 alpha
IRF6: interferon regulatory factor 6
PGC1α: peroxisome proliferator-activated receptor gamma coactivator 1-alpha
RORα: RAR-related orphan receptor alpha
MoMFs: pro-inflammatory monocytes
MASH: metabolic dysfunction-associated steatohepatitis
CD68: cluster of differentiation 68
MARCO: macrophage receptor with collagenous structure
SR-A: scavenger receptor class A
KCs: Kupffer cells
NK: natural killer cells
γδ T cells: gamma delta T cells
IL-15: interleukin-15
NKG2D: natural killer group 2D
DNAM-1: DNAX accessory molecule-1
NKp30: natural cytotoxicity triggering receptor 3
MCP-1: monocyte chemoattractant protein-1
F4/80: EGF-like module-containing mucin-like hormone receptor-like 1
CD80: cluster of differentiation 80
CD163: cluster of differentiation 163
CD206: cluster of differentiation 206
PGC-1α: Peroxisome proliferator-activated receptor gamma coactivator 1-alpha
KLF4: Krüppel-like factor 4
CD86: cluster of differentiation 86
SMAD: mothers against decapentaplegic homolog
AMPK: AMP-activated protein kinase
G6PD: glucose-6-phosphate dehydrogenase
JAK2: Janus kinase 2
STAT3: signal transducer and activator of transcription 3
NLRP3: NACHT, LRR and PYD domains-containing protein 3
FGF21: fibroblast growth factor 21
HO-1: heme oxygenase-1
GPX4: glutathione peroxidase 4
IGF-1: insulin-like growth factor 1
mTOR: mechanistic target of rapamycin
CX3CL1: C-X3-C motif chemokine ligand 1
CaMKII: Ca²⁺/calmodulin-dependent protein kinase II
CX3CR1: C-X3-C motif chemokine receptor 1
S/E: sedentary ethanol
RCR: respiratory control ratio
P:O: phosphorous-to-oxygen ratio
GSH: Glutathione
CLD: chronic liver disease
HSCs: hepatic stellate cells
CCN1: cellular communication network factor 1
TGF-β: transforming growth factor beta
mTORC1: mechanistic target of rapamycin complex 1
Akt: protein kinase B
S6-RP: ribosomal protein S6
p53: tumor protein p53
p27: cyclin-dependent kinase inhibitor 1B
CDK2: cyclin-dependent kinase 2
JNK1: c-Jun N-terminal kinase 1
AP-1: activator protein 1
PTEN: phosphatase and tensin homolog
CD31: cluster of differentiation 31
TP53: tumor protein p53
β-catenin: beta-catenin
AICAR: 5-aminoimidazole-4-carboxamide ribonucleotide
TZDs: thiazolidinediones
MASLD/MASH: metabolic dysfunction-associated steatotic liver disease/metabolic dysfunction-associated steatohepatitis
MOTS-c: mitochondrial open reading frame of the 12S rRNA-c
Smad2/3: intracellular signaling proteins
